# Psychometric evaluation of the Major Depression Inventory (MDI) as depression severity scale using the LEAD (Longitudinal Expert Assessment of All Data) as index of validity

**DOI:** 10.1186/s12888-015-0529-3

**Published:** 2015-08-05

**Authors:** Per Bech, N. Timmerby, K. Martiny, M. Lunde, S. Soendergaard

**Affiliations:** Psychiatric Research Unit, Psychiatric Centre North Zealand, Copenhagen University Hospital, Dyrehavevej 48, DK-3400 Hillerød, Denmark; Intensive outpatient unit for Affective Disorders (IAA), Psychiatric Centre Copenhagen, Rigshospitalet, Copenhagen University Hospital, Copenhagen, Denmark

**Keywords:** LEAD, Standardization, Major depression inventory, Hamilton depression scale, Melancholia scale, Visual analogue scale

## Abstract

**Background:**

The Major Depression Inventory (MDI) was developed to cover the universe of depressive symptoms in DSM-IV major depression as well as in ICD-10 mild, moderate, and severe depression. The objective of this study was to evaluate the standardization of the MDI as a depression severity scale using the Visual Analogue Scale (VAS) as index of external validity in accordance with the LEAD approach (Longitudinal Expert Assessment of All Data).

**Methods:**

We used data from two previously published studies in which the patients had a MINI Neuropsychiatric Interview verified diagnosis of DSM-IV major depression. The conventional VAS scores for no, mild, moderate, and severe depression were used for the standardization of the MDI.

**Results:**

The inter-correlation for the MDI with the clinician ratings (VAS, MES, HAM-D_17_ and HAM-D_6_) increased over the rating weeks in terms of Pearson coefficients. After nine weeks of therapy the coefficient ranged from 0.74 to 0.83.

Using the clinician-rated VAS depression severity scale, the conventional MDI cut-off scores for no or doubtful depression, and for mild, moderate and severe depression were confirmed.

**Conclusions:**

Using the VAS as index of external, clinical validity, the standardization of the MDI as a measure of depression severity was accepted, with an MDI cut-off score of 21 for mild depression, 26 for moderate depression severity, and 31 for severe depression.

**Trial registration:**

Martiny et al. Acta Psychiatr Scand 112:117-25, 2005: None – due to trial commencement date.

Straaso et al. Acta Neuropsychiatr 26:272-9; 2014: ClinicalTrials.gov ID NCT01353092.

## Background

The Major Depression Inventory (MDI) was developed [1, 2] to cover the universe of depressive symptoms in DSM-IV major depression [3] and in ICD-10 depression [4] (mild, moderate, severe). Consequently the time frame (window) for the MDI is the past two weeks to accord with DSM-IV and ICD-10.

The MDI can be used as a diagnostic scale by following the algorithms in accordance with DSM-IV or ICD-10. Using as index of diagnostic validity the Schedules for Clinical Assessment in Neuropsychiatry (SCAN) [5] administered by experienced psychiatrists, we obtained a sensitivity of 90 % and a specificity of 82 % for DSM-IV major depression [5].

Via its summed total score the MDI can also be a measure of depression severity analogue to the Zung Self-rating Depression Scale (Zung-SDS, [6]) or the Beck Depression Inventory (BDI, [7]). However, we have previously shown that the MDI is superior to the Zung-SDS ([1] and to the BDI [8]. Another widely used depression questionnaire, the Patient Health Questionnaire (PHQ-9) [9], was developed with reference to DSM-IV. However, the PHQ-9 was especially constructed to capture the diagnosis of major depression, not to be a measure of depression severity like the BDI. In contrast, the MDI actually fulfils both Mokken’s non-parametric item response theory model [10] and Rasch’s one-parametric model [11] as shown by Olsen et al. [5] and can thus be used as a unidimensional depression severity scale. However, we still need to confirm the conventional cut-off scores of MDI, such as that of >25 for major depression.

The clinical validity of a scale must be evaluated by the use of an independent global severity assessment performed by an experienced clinician. Spitzer [12] called this procedure the LEAD (Longitudinal Expert Assessment of All Data) approach. By “expert” Spitzer [12] was referring to a clinician who had demonstrated his or her competence to make this assessment based on a thorough clinical interview taking all available data into account. This LEAD approach was used in our validation study of the Hamilton Depression Scale [13] and was also used by Maier [14] when he validated the Hamilton Scale (HAM-D_17_), the Montgomery Åsberg Depression Rating Scale (MADRS) [15] and the Bech-Rafaelsen Melancholia Scale (MES) [2]. In the analysis to be reported here we used a Visual Analogue Scale (VAS) from 0 to 100 mm for the LEAD assessment of depression severity [16, 17].

The objective of this study was to evaluate the MDI as a depression severity scale using both a global VAS assessment as well as the Hamilton Depression Scale (HAM-D_17_) and the Bech-Rafaelsen Melancholia Scale (MES) as indices of external validity.

## Methods

Patients: we have used data from two previously published studies in which weekly ratings were performed:

- Study 1: Martiny et al. [18]

A randomised, double-blind trial with bright light therapy versus sham light therapy as adjunct treatment to sertraline in non-seasonal major depression. In total, 102 patients with DSM-IV major depression, as verified by the Mini International Neuropsychiatric Interview (MINI) [19], were included. The planned duration of this trial was 9 weeks (with 5 weeks of the adjunct treatment and a follow-up four weeks later); in total, therefore, seven rating occasions to be analysed.

Ethics: The study was carried out according to the declaration of Helsinki and the ICH-GCP guidelines (International Conference on Harmonisation, Good Clinical Practice). The study was approved by the Ethical Committee for the Counties of Bornholm, Frederiksborg, Roskilde and Storstrøm and by the Danish Central Data Register. Patients were given information as requested by the Biomedical Research Ethics, and all patients signed an informed consent.

- Study 2: Straaso et al. [20]

A randomised, double-blind controlled dose-remission study with pulsating electromagnetic fields as augmentation in therapy-resistant depression. In total, 65 patients with DSM-IV major depression, as verified by the MINI [19] were included. The planned duration of this trial was 9 weeks (with 8 weeks of pulsating electromagnetic fields therapy as augmentation and a follow-up one week later). In order to balance with the Study 1 ratings we have focused on the first five weeks and the last week, therefore in total seven rating occasions were analysed.

The study was carried out in accordance with the Declarations of Helsinki and the European Union directive of Good Clinical Practice. The study was approved by the Danish Health and Medicines Authority (2013030959) and the Committee on Biomedical Research Ethics (H-1-2010-031) and was reported to the Danish Data Protection Agency (PSV-2010-2). The trial was registered at ClinicalTrials.gov (ID NCT01353092). Patients were given information as requested by the Biomedical Research Ethics, and all patients signed an informed consent.

### Psychometrics

In the present analysis we have focussed on the following clinician-administrated rating scales:

The Hamilton Depression Scale (HAM-D_17_) in combination with the Melancholia Scale (MES) with a scoring sheet [16, 17] in which a Visual Analogue Scale for Depression Severity (VAS) is placed at the bottom as a horizontal line from 0 (no depression) to 100 mm (extreme depression). The interviewer is asked to score the VAS before completing the HAM-D_17_ and MES. The LEAD procedure (Longitudinal Expert Assessment of All Data) was thus used to make the global severity assessment of depressive states taking into account all available data over the past three days.

As discussed elsewhere [17] the horizontal version (yard stick-line) with descriptive cues at each end and 100 mm in between is generally preferred.

The LEAD principle was used to clinically validate the HAM-D_17_ [13] which resulted in that six of the Hamilton items (depressed mood, guilt feelings, work and interests, psychomotor retardation, psychic anxiety, and general somatics (fatigability)), HAM-D_6_, were found to be most valid when associated with experienced psychiatrists’ global assessment of depression severity. The Bech-Rafaelsen Melancholia Scale (MES) was developed to capture the six HAM-D_6_ core items with reference to the Cronholm-Ottosson Depression Scale [21]. For a review of the MES, see [22].

The three depression symptom rating scales (HAM-D_17_, HAM-D_6_, MES) were rated on a weekly basis by KM and ML, as was the VAS, using the time frame of the past three days for the VAS as well. The MDI was also completed each week by the patients. The clinicians (KM, ML) had no access to the MDI scorings. The inter-rater reliability of KM and ML as Danish University Antidepressant Group (DUAG) raters has been found acceptable with intraclass coefficients of 0.89 (HAM-D_6_), 0.93 (HAM-D_17_) and 0.91 (MES) [Martiny et al.: Relapse prevention in major depressive disorder: A four-arm randomised 6-month double-blind comparison of three fixed dosages of escitalopram and a fixed dose of nortriptyline in patients successfully treated with acute electroconvulsive treatment (DUAG-7) – Submitted 2015].

#### The Major Depression Inventory (MDI)

In the studies analysed in this report the time frame of the MDI was the past week and not the conventional two weeks, due to the fact that the MDI was used at weekly rating sessions in the two trials.

**Fig. 1:**
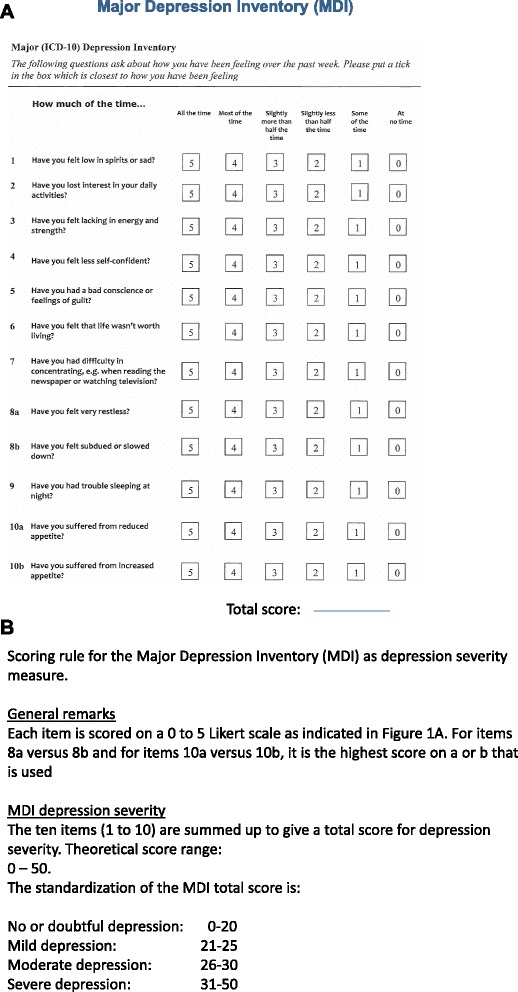
**a** The Major Depression Inventory (MDI) questionnaire with the time frame of one week. **b** Scoring rule for the Major Depression Inventory (MDI) as depression severity measure

Figure 1a shows the Major Depression Inventory (MDI) with the time frame of one week while Fig. 1b shows how this questionnaire can be used both as a depression severity scale by its total scale score from 0 = no depression to 50 = extreme depression, and as a diagnostic scale following the algorithm of ICD-10 depression [4] or of DSM-IV major depression [3]. When using the MDI as a depression severity scale by its total score we have, as shown in Fig. 1b, suggested the cut-off scores for no, doubtful, mild, moderate, and severe depression.

### Statistical analysis

We used the SAS statistical package (version 9.0.0, 2002) both for the proportion of variance of the dependent variable (VAS) that is accounted for by the independent variable (MDI) within a regression analysis using R^2^ > 0.50 as goodness of fit [23] and for the intercorrelations between the depression scales in terms of Pearson coefficients [24]. The weighted Kappa was used when testing the corresponding cut-off points between VAS and MDI [25].

## Results

Table 1 shows the age, gender, and HAM-D_17_ baseline mean score in study 1 and study 2.

**Table 1:** Age, gender, and HAM-D17 baseline mean score in Study 1 and Study 2

	Study 1		Study 2	
	Martiny et al. 2005 [18]		Straaso et al. 2014	
	All included patients *N* = 102	Patients with complete ratings at the seven rating weeks *N* = 70	All included patients *N* = 65	Patients with complete ratings at the seven rating weeks *N* = 48
Age, years, mean (sd)	44.7 (16.1)	45.1 (14.9)	48.1 (12.7)	47.6 (12.1)
Gender % females	70	75	63	63
Baseline HAM-D_17_, mean (sd)	22.3 (3.9)	22.4 (3.3)	20.6 (2.8)	20.6 (3.0)

In study 1 a total of 70 patients had complete scorings on all the included weeks. In study 2 a total of 48 patients had complete scorings. Thus 118 patients, or 70 % of the 150 patients included in the two studies, were analysed.

Table 2 shows the inter-correlation of the MDI total score with VAS, MES, HAM-D_17_, and HAM-D_6_ from baseline to week 8 for the 118 patients, i.e. seven rating weeks in total. At the bottom in Table 2 all seven rating weeks with 826 observations are also shown. The association between the MDI and VAS in terms of Pearson coefficients was generally lower than the association between the MDI and MES, HAM-D_17_ and HAM-D_6_. After two weeks of therapy the four clinician-administered scales obtained a Pearson coefficient of 0.60 or higher when correlated to MDI. Taking all weeks into consideration (*N* = 826), a Spearman coefficient of 0.70 or higher was obtained (Table 2).

**Table 2:** Pearson inter-correlation for the MDI at the various weeks of treatment (*N* = 118)

Weeks	VAS	MES	HAM-D_17_	HAM-D_6_
Baseline	0.39	0.54	0.51	0.32
Week 1	0.45	0.66	0.61	0.57
Week 2	0.64	0.73	0.72	0.70
Week 3	0.61	0.74	0.73	0.72
Week 4	0.69	0.79	0.77	0.76
Week 5	0.67	0.80	0.79	0.76
Week 9	0.66	0.84	0.83	0.82
All weeks (*N* = 826)	0.74	0.83	0.82	0.80

**Fig. 2:**
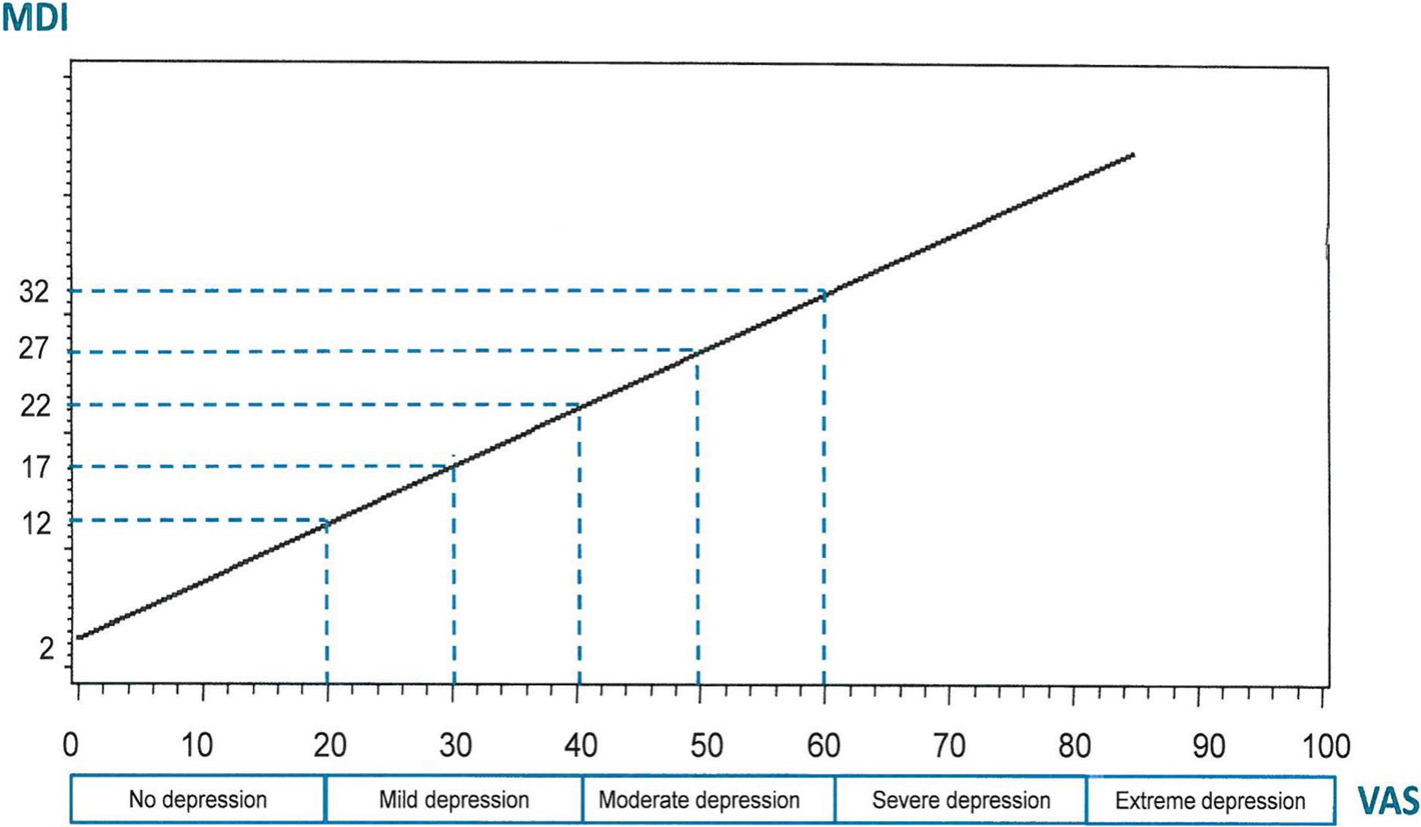
Regression analysis using the VAS scores to arrive at the corresponding MDI scores by the formula MDI = 0.49 x VAS + 2.40 (*N* = 826)

Figure 2 shows the regression analysis using the VAS scores to arrive at the corresponding MDI scores by the formula MDI = 0.49 x VAS + 2.40 (*N* = 826). The R^2^ was 0.55, indicating an acceptable goodness of fit. As indicated at the abscissa in Fig. 2 a VAS score of 50 is the average cut-off in moderate or major depression. Using the regression formula, a VAS score of 50 corresponds to a MDI score of 26.9 which is rather similar to the conventional MDI cut-off score of ≥ 26 (Fig. 1b). Using the regression formula, a VAS score of 60 corresponds to a MDI score of 31.8, which is rather similar to the conventional MDI cut-off score of ≥ 31 for severe depression (Fig. 1b). Similarly, a VAS score of 40 corresponds to a MDI score of 22.0, this is quite close to the conventional MDI cut-off ≥ 21 for mild depression (Fig. 1b). Finally, a VAS score of 30 corresponds to a MDI score of 17.1, this is rather close to the conventional MDI cut-off score of < 16 for no or doubtful depression (Fig. 1b).

When using the MDI cut-off scores of 0–20, 21–25, and >25 versus VAS cut-off scores of 0–40, 41–50, and > 50, the distribution of the 826 observations was not random (weighted Kappa was 0.49, *P* < 0.001).

When using the conventional HAM-D_17_ cut-off score of 18 for major depression and the MDI cut-off score of > 25, we found that within the 826 observations (Table 2) the percentage convergence of MDI was 156 out of 195 observations with HAM-D_17_, or 80.0 %, i.e. an acceptable convergence, but of moderate degree.

Concerning the MDI algorithm for DSM-IV major depression or ICD-10 depression, we used the MINI diagnoses at baseline, excluding the observations with low HAM-D_17_ scores between 13 and 18 (*N* = 97). The MDI algorithm for DSM-IV depression identified 72 of the 97 patients, or 74.2 %. The MDI algorithm for ICD-10 depression identified 76 of the 97 patients, or 78.3 %.

## Discussion

In the data set analysed in this report the MDI was used as an outcome scale at the weekly ratings during a planned treatment period of nine weeks covering seven rating occasions. In this situation the MDI time frame was the past week and not the past two weeks as conventionally applied when the MDI is included as a diagnostic tool with reference to DSM-IV or ICD-10.

Using the clinician-rated VAS depression severity scale, the conventional cut-off standardization for no or doubtful depression, and for mild, moderate and severe depression was confirmed.

However, when pooling all assessments (*N* = 826), we actually introduce a mixture of both inter-individual differences and intra-individual changes as the patients are included at the various rating occasions. On the other hand, this mixed effects model approach has had a very slight influence in our analysis.

The reason for the moderate Pearson coefficients at the baseline ratings is that the score range on the various scales at that point in time is rather limited because the patients had to be in a depressive state and in need of therapy at inclusion in the two studies [1, 2].

The MDI cut-off score of >25 for major depression had a percentage convergence of 80 % with the HAM-D_17_ score of >18. The MDI cut-off point of > 25 has been found acceptable both in a sample of psychiatric outpatients with affective disorders [26] and in a general population sample when compared to patients with a first episode of psychotic depression followed up over 6 years [27].

A self-rating scale rather similar to the MDI is the Patient Health Questionnaire (PHQ-9) which was originally developed to screen for depression in primary care [10]. The PHQ-9 is defined by the DSM-IV symptoms of depression and thus not designed for ICD-10 depression. However, the quantifier of the individual items differs from the MDI. Zimmerman [28] has evaluated the role of the PHQ-9 in connection with the need for a DSM-5 self-rating questionnaire to measure the dimensional approach to major depression. Zimmerman [28] has in this respect shown that the standardization of the PHQ-9 is not based on empirical studies, and that the conventionally used cut-off score overestimates the prevalence of depression when using the Hamilton Depression Scales as index of validity. Moreover, an analysis using the item response theory formulated by Rasch, Forkmann et al. [29] showed that the summed total score is not a sufficient statistic as a measure of depression severity. This is a conditio sine qua non for using the total score as cut-off index in the diagnosis of major depression. As recommended by Forkmann et al. [29] the diagnostic algorithm for DSM-IV major depression should be used in connection with the PHQ-9. The DSM-5 major depression diagnosis has maintained the same symptom universe and the same diagnostic algorithm as the DSM-IV. In this respect the recommendation put forward by Forkman et al. [29] is still valid for PHQ-9 in the DSM-5 context as is the MDI for the DSM-5 major depression diagnosis. Furthermore, the MDI has been accepted by the Rasch model [13] as a unidimensional scale for depression severity, which is the background for the standardization analysis performed in this report.

A limitation of this analysis is that we have used the time frame covering the past week and not the conventional frame of two weeks. On the other hand we have focused on the standardization of the MDI when used as a depression severity measure rather than when used for diagnostic properties. Another limitation is that completed data for all the ratings was not available for all the patients included in the two trials under examination. On the other hand, a coverage of 70 % as obtained in this analysis is acceptable in clinical trials of depression [30].

## Conclusions

The clinical validity of the MDI as a unidimensional depression severity scale has been found acceptable using the global clinical VAS scale performed by experienced clinicians as index of validity. The conventional standardization of the MDI with cut-off scores for no, mild, moderate, and severe depression has been found adequate.
